# Antivivisection Redivivus

**Published:** 1900-02

**Authors:** W. W. Keen

**Affiliations:** President American Medical Association


### ANTIVIVISECTION REDIVIVUS.

THE antivivisectionists are again on the warpath led by that old battle-scared veteran Senator Gallinger, of New Hampshire. He has marched up the hill and marched down again several times before in similar campaigns, and we hope he may be put to rout so completely this time that there will not be left "a shirt and a half in all his company."

There is, however, a very serious danger threatening the interests of experimental medicine and all studies of diseases of the lower animals. The antivivisectionists are resorting to every subterfuge to carry their pet bill through congress. They are appealing to the sympathy of tender-hearted men and women, are uniting their forces in strong aggressive opposition to vivisection, and are growing more determined year by year. They publish books, periodicals and brochures on the subject, which they distribute broadcast with a lavish expenditure. Even novels and storiettes are written with a view to prejudice or influence the weak or indifferent, Mrs. Elizabeth Stuart Phelps-Dodge having lately contributed a little of her "Loveliness" to the cause. Her story, however, is so improbable on its face that it will tend to strengthen rather than weaken the side of our inclining.

Dr. Charles E. Congdon, of this city, very opportunely invited attention to this subject at the recent meeting of the Medical Society of the County of Erie, asking the society to array itself in opposition to Senator Gallinger's bill. The legislative committee was empowered to oppose the measure according to its discretion.

The president of the American Medical Association, Dr. W. W. Keen, of Philadelphia, has addressed an open letter to the profession, which sets forth the dangers and importance of the situation and suggests definite action. We take pleasure in presenting it in full as a part of this appeal to our readers to take steps at once looking to the defeat of this unscientific and unhumane piece of legislation. It is as follows:

TO THE MEMBERS OF THE MEDICAL PROFESSION IN THE UNITED STATES :

THE cause of humanity and of scientific progress is seriously menaced. Senator Gallinger has again introduced into Congress the Bill for the "Further Prevention of Cruelty of Animals in the District of Columbia," which he has so strenuously and misguidedly advocated in the last two Congresses. It is Senate Bill No. 34. Twice the Committee on the District of Columbia has, also unfortunately and misguidedly, reported the bill with a favorable consideration. It is speciously drawn to seem as if it were intended only in the interest of prevention of cruelty to animals, but the real object is twofold : 1, to prohibit vivisection and, 2, to aid the passage of similar bills in all the state legislatures.

It hardly needs to be pointed out that this would seriously interfere with or even absolutely stop the experimental work of the Bureau of Animal Industry and the three medical departments of the Government, the Army, the Navy, and the Marine Hospital Service. The animals themselves might well cry out to be saved from their friends. No more humane work can be done than to discover the means of the prevention of diseases which have ravaged our flocks and herds. All those who raise or own animals, such as horses, cattle, sheep, pigs, chickens, etc., are vitally interested in the preservation of their health and the prevention of disease.

The inestimable value of these scientific researches as to the prevention and care of disease among hnman beings it is superfluous to point out. Modern surgery and the antitoxin treatment of diphtheria alone would justify all the vivisection ever done.

As my attention has been called officially to the introduction of the bill, I take the opportunity of appealing to the entire profession of the country to exert itself to the utmost to defeat this most cruel and inhuman effort to promote human and animal misery and death and to restrict scientific research. It is of the utmost importance that *every physician* who shall read this appeal shall *immediately* communicate especially with the senators from his state, shall also invoke the aid of the representatives from his or other districts in his state, and by vigorous personal efforts shall aid in defeating the bill.

It is especially requested also that all of the national, state and county societies, at their next meeting, take action looking toward the same end. If regular meetings are not soon to be held, special meetings should be called. Correspondence is invited from all those who can give any aid.

The Committee on the District of Columbia consists of Senator James McMillan, Michigan, Chairman, and Senators J. H. Gallinger New Hampshire; H. C. Hansborough, North Dakota; Redfield Proctor, Vermont; J. C. Pritchard, North Carolina; Lucien Baker, Kansas; C. P. Wetmore, Rhode Island; C. J. Faulkner, West Virginia; Thomas S. Martin, Virginia; Wm. M. Stewart, Nevada; and Richard Kenney, Delaware. Personal letters may be addressed to them or to other senators. Petitions should be addressed to the Senate of the United States.

W. W. Keen, M. D.
President American Medical Association.

